# Primed to be strong, primed to be fast: modeling benefits of microbial stress responses

**DOI:** 10.1093/femsec/fiz114

**Published:** 2019-07-11

**Authors:** Felix Wesener, Britta Tietjen

**Affiliations:** 1 Freie Universität Berlin, Institute of Biology, Biodiversity/Theoretical Ecology Group, Altensteinstraße 34, 14195 Berlin, Germany; 2 Berlin Brandenburg Institute of Advanced Biodiversity Research, 14195 Berlin, Germany

**Keywords:** stress ecology, adaptive defense, primed response patterns, cost–benefit analysis, population dynamics, community ecology

## Abstract

Organisms are prone to different stressors and have evolved various defense mechanisms. One such defense mechanism is priming, where a mild preceding stress prepares the organism toward an improved stress response. This improved response can strongly vary, and primed organisms have been found to respond with one of three response strategies: a shorter delay to stress, a faster buildup of their response or a more intense response. However, a universal comparative assessment, which response is superior under a given environmental setting, is missing. We investigate the benefits of the three improved responses for microorganisms with an ordinary differential equation model, simulating the impact of an external stress on a microbial population that is either naïve or primed. We systematically assess the resulting population performance for different costs associated with priming and stress conditions. Our results show that independent of stress type and priming costs, the stronger primed response is most beneficial for longer stress phases, while the faster and earlier responses increase population performance and survival probability under short stresses. Competition increases priming benefits and promotes the early stress response. This dependence on the ecological context highlights the importance of including primed response strategies into microbial stress ecology.

## Teaser

Using mathematical simulation models, the benefit of different microbial priming strategies is examined under various environmental conditions.

## INTRODUCTION

Microorganisms are subject to stressors of different nature and intensity, and have thus developed various response mechanisms to counteract these stressors. In contrast to constitutive stress defenses, which are always expressed, an induced direct defense is often activated directly upon the encounter of an initial stress. This initial, often milder, stress does not necessarily immediately initiate an active stress defense by, for example, inducing the production of defense molecules. Instead, it can lead to a more efficient defense only upon the occurrence of stronger environmental stress, which has been termed ‘priming’ (Hilker *et al*. 2016). Alternative terms for priming include ‘stress hardening’ (Lou and Yousef 1997), ‘acquired stress response’ (Berry and Gasch 2008; Guan *et al*. 2012) or ‘cross protection’, if a mild stress confers enhanced resistance toward a stressor of a different nature (Rangel, Anderson and Roberts 2008; Dhar *et al*. 2013; Hilker *et al*. 2016). Priming has been found in plants (Baldwin and Schmelz 1996; Newman *et al*. 2002; Hulten *et al*. 2006; Pozo *et al*. 2009), in the mammalian immune system (Gifford and Lohmann-Matthes 1987; Hayes and Zoon 1993; Hayes, Freeman and Donnelly 1995) and in different groups of microbes such as bacteria (Koutsoumanis and Sofos 2004; Mitchell *et al*. 2009; Cebrián *et al*. 2010; Hernández *et al*. 2012; Andrade-Linares, Lehmann and Rillig 2016), fungi (Alvarez-Peral *et al*. 2002; Berry and Gasch 2008; Rangel, Anderson and Roberts 2008; Mitchell *et al*. 2009; Andrade-Linares, Veresoglou and Rillig 2016), and archaea (Trent 1996).

Priming can be a cost-saving strategy in fluctuating, but predictive environments, since the environmental cue (also called priming stimulus) does not require the full commitment of a direct induced defense, but instead improves the defense against a possible future stress. When assessing the effectiveness of an induced stress defense, the cost–benefit ratio of this response can be used as a measure of success, since a certain behavior or physiological process can only be evolutionary persistent if it confers benefits that are higher than the invested costs. The costs of priming have been studied in plants (Hulten *et al*. 2006) and animals (Krebs and Loeschcke 1994); however, studies on priming costs in microbes are missing and the molecular basis of priming is poorly understood. In yeast, the genes activated after a mild oxidative stress only partly overlap the genes of direct defense (Kelley and Ideker 2009), indicating distinct mechanisms. However, the mechanisms of priming vary greatly not only between taxa, but also within a single organism: For example, priming for ${\mathrm{ H}_2}{\mathrm{ O}_2}$ tolerance in yeast involves different sets of genes depending on the nature of the priming stimulus (Berry *et al*. 2011). Since priming is not the result of one universal molecular or physiological process, also the ecologically observed response of an organism to an impending stress can strongly differ. In the ecological context, it is therefore essential to evaluate the impact of different priming responses on the performance of an organism or population. Four different improved stress responses of a primed organism were described, namely a stronger, a faster, an earlier and a more sensitive response than a naïve organism (Conrath *et al*. 2006, Hilker *et al*. 2016). In the following, we will focus on faster, earlier and stronger primed responses and will briefly introduce the three responses jointly with potential underlying mechanisms at the molecular level.

An ‘earlier’ response would exhibit the same kinetics as a naïve stress response with an induced direct defense, but with a shorter lag phase until the stress response starts to build up. Therefore, the final defense level will be reached earlier than in the naïve state. Possible underlying molecular mechanisms of the earlier primed response could, for example, be based on the accumulation of transcription factors due to a previous priming stimulus leading to an earlier start of transcription and translation of response proteins after a triggering stress. Primed yeast cells, for example, have been shown to react earlier to sudden exposure to fungicidal stress due to predictive translation and transcription (Berry and Gasch 2008). The dynamics of the ‘faster’ stress response are characterized by a similar lag phase as the naïve response but a steeper slope in the stress defense buildup. This response could be caused by hyperactivation and a faster signaling cascade, leading to a faster buildup of the stress defense. For example, cells of *Saccharomyces cerevisiae* that were repeatedly exposed to NaCl exhibited faster gene expression if exposed to ${\mathrm{ H}_2}{\mathrm{ O}_2}$ afterward (Guan *et al*. 2012). A ‘stronger’ stress response initially resembles the naïve response (exhibiting the same lag phase and slope) but eventually reaches a higher final response level than the earlier, the faster and the naïve response. Here, too, hyperactivation and an enhanced gene expression could be responsible and lead to a higher response amplitude. *Bacillus subtilis*, for example, showed a significantly increased survival during heat stress of 52°C when primed with a 48°C heat shock beforehand, caused by raised levels of the Spx transcription factor (Runde *et al*. 2014).

As we observe all three proposed primed response types in nature, the question arises, which response could be most beneficial for an organism. In a systematic analysis of costs and benefits of different priming response strategies, Douma *et al*. (2017) addressed this question for a single plant organism suffering from herbivory. However, a universal analysis for microbial populations and communities is still missing. Here, we examine the benefits of the three priming responses in a highly generalized ordinary differential equation (ODE) model that describes the performance of primed and unprimed microbial populations under stress. Since we expect the benefits of the priming strategies to be highly context dependent, we use the ODE model to quantify the effect of the priming response strategies on population performance under different stress durations and priming costs for species in isolation and in a community, as well as the efficiency of these strategies in preventing extinction of a population.

## METHODS

We used a descriptive ODE model simulating the population size of an arbitrary microbial species to investigate which of three potential primed stress responses (faster, earlier or stronger) is most beneficial compared to a naïve stress response. We assessed different stress conditions and priming costs to determine how these factors affect the different primed response types.

### Model description

The model describes the dynamics of a microbial population (measured in terms of biomass or colony forming units) growing in isolation and later in competition with constant, but limited resources. The population experiences a triggering stress of a given duration $\mathrm{ TD}$, beginning at a certain point in time ${t_{\mathrm{ TS}}}$. We chose stress dynamics to occur at the same timescale as population dynamics, which can range from minutes to days or weeks, and thus refer to a general time unit *t*. We used the relative difference in size between primed and naïve populations as direct measure of population performance. Thus, we could assess the effectiveness of primeability under different conditions by comparing the size of a primed population with the size of a naïve population after the triggering stress event has ended at $t\ = {t_{\mathrm{ TE}}}\ $.

The basic model describes a simple exponential function of population size ($S$) at time ($t$). The growth model was extended by functions describing the growth rate ($g( {P},{ t} )$) dependent on impacts of priming ($P$) events at a given point of time and an additional mortality rate ($m( {T,t} )$):

(1) \begin{eqnarray*}
\frac{{\mathrm{ d}S}}{{\mathrm{ d}t}} = g\left( {P,t} \right) \cdot S\left( t \right)\ - m\left( {T,t} \right) \cdot S\left( t \right).
\end{eqnarray*}

The costs of priming appear as reduced growth rate during the priming phase for a primeable population, while the naïve population does not exhibit a reduction in growth during this phase. This cost factor reflects additional transcription and translation that are induced by priming and are expected to exert a constant cost rate (Stoebel, Dean and Dykhuizen 2008) leading to costs proportional to growth (Mitchell and Pilpel 2011). The subsequent triggering stress is applied directly after the priming phase. Here, we defined adversary effects on microbial populations as disturbance that leads to partial or total destruction of biomass and therefore implemented the triggering stress as additional mortality rate $m( {T,t} )$ while the stress is lasting (for duration $\mathrm{ TD}$). However, triggering does not impact the intrinsic growth rate.

(2) \begin{eqnarray*}
g\ \left( {P,t} \right) = \left\{ {\begin{array}{@{}*{2}{l}@{}} {{g_\mathrm{ I}},}&{t < {t_\mathrm{ P}},}\\ {{g_\mathrm{ I}} \cdot \left( {1 - {c_\mathrm{ P}}} \right),}&{{t_\mathrm{ P}} \le t < {t_{\mathrm{ TS}}},}\\ {{g_\mathrm{ I}},}&{t \ge {t_{\mathrm{ TS}}}.} \end{array}} \right.
\end{eqnarray*}

A primeable species exhibits a growth rate reduced by ${c_\mathrm{ P}}$ during the priming phase. Since we assumed the priming stress to be mild, the growth of a nonprimeable species remains at its initial level during this period, i.e. ${c_\mathrm{ P}} = \ 0.\ $ At the beginning of the triggering stress at time ${t_{\mathrm{ TS}}}$, the priming costs ${c_\mathrm{ P}}$ are set back to zero, because we assumed the stress defense costs to be equal between naïve and primed response.

(3) \begin{eqnarray*}
m(T,t) = \left\lbrace {\begin{array}{ll} 0,& \quad t \,\lt\, t_{\mathrm{ TS}},\\
m_I,& \quad t_{\mathrm{ TS}} \le t \,\lt\, t_L,\\
(- {s_R} \cdot (t - t_L) + 1) \cdot m_I, & \quad t_L \le t \,\lt\, t_R,\\
m_R, &\quad t_R \le t \le t_{\mathrm{ TE}}.\end{array}}\right.
\end{eqnarray*}

A triggering stress instantly leads to an initial high mortality ${m_\mathrm{ I}}$ for the duration of a lag phase $L$, since the organism needs to induce a stress response to counteract the stressor. At time point ${t_\mathrm{ L}}$, the stress response starts building up and linearly reduces the mortality rate with response speed ${s_\mathrm{ R}}$ until the maximum response level with mortality ${m_\mathrm{ R}}$ is reached, which is not further improved as long as the stress lasts (until ${t_{\mathrm{ TE}}}$). A primed organism is assumed to exhibit an improved response to the triggering stress, which is, as proposed by Hilker *et al*. (2016), realized by an earlier, a faster or a stronger stress response. We did not evaluate the additionally proposed more sensitive response, as sensitivity cannot be quantitatively investigated in a similar manner as the other response types without modulating the stress intensity. When the stress vanishes (after stress duration $\mathrm{ TD}$ at time point ${t_{\mathrm{ TE}}}$), we used population size $S( {t\ = {t_{\mathrm{ TE}}}\ } )$ as estimation of population fitness and calculate the relative benefits of priming as the relative difference to the population size of a naïve population. Note that in our model, mortality is never lower than the primed growth rate ${m_\mathrm{ I}} > {g_\mathrm{ I}} \cdot ( {1 - {c_\mathrm{ P}}} )$, which always results in a negative effect of ${m_\mathrm{ I}}$.

### Baseline scenario for primed responses

**Figure 1.**
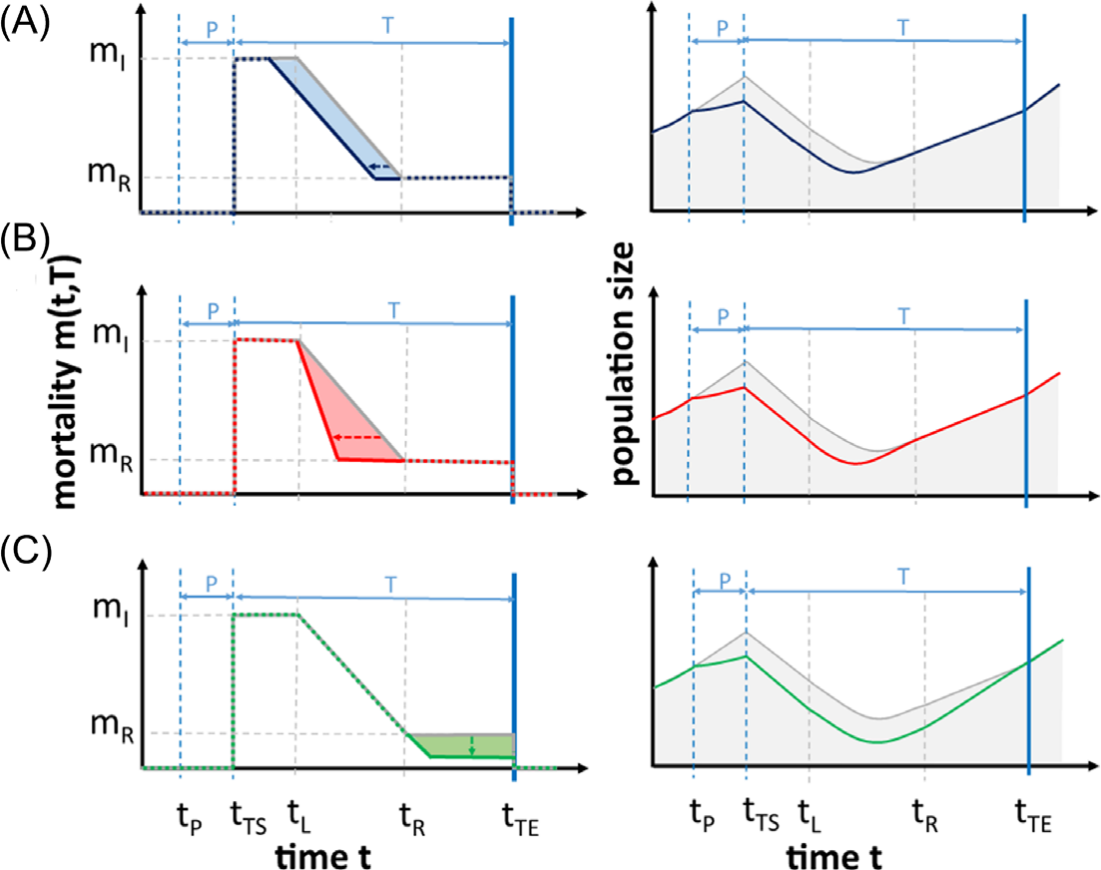
Potential responses of primed and naïve species toward stress impacts on mortality and the resulting population size. Left panels: Mortality of primed and naïve species, having (**A**) a stronger, (**B**) an earlier and (**C**) a faster response. Right panels: the respective population dynamics. In all panels, the naïve stress response is represented by the gray line, and the colored line represents the primed response. Priming costs are not illustrated here, as they do not affect mortality. Abbreviations: $P\ $ = priming; $T\ $ = triggering; ${m_\mathrm{ I}}$ = initial mortality; ${m_\mathrm{ R}}$ = final mortality level of the stress response; ${t_\mathrm{ P}}$ = beginning of the priming phase; ${t_{\mathrm{ TS}}}$ = beginning of the triggering stress; ${t_\mathrm{ L}}$ = end of the response lag phase; ${t_\mathrm{ R}}$ = time point when ${m_\mathrm{ R}}$ has been reached; ${t_{\mathrm{ TE}}}$ = end of triggering stress.

The three primed response types were realized as follows: an ‘earlier’ response leads to a shorter lag duration $L$ and thus a lower value of ${t_\mathrm{ L}}$ in comparison to a naïve organism (Fig. 1A). The ‘faster’ stress response is characterized by a higher absolute value of the slope ${s_\mathrm{ R}}$, which causes a steeper slope in the decrease of the mortality rate (Fig. 1B) compared to the response of the naïve organism. A ‘stronger’ stress response modulates the final response value ${m_\mathrm{ R}}$ (Fig. 1C) that can be reached while the stress lasts.

To allow for direct comparison between the three response types, we defined a baseline stress scenario. In this scenario, the specific primed response parameters ${L_\mathrm{ p}}$, ${s_{\mathrm{ Rp}}}$ or ${m_{\mathrm{ Rp}}}$, respectively, were adjusted in a way that the benefits of priming exactly compensate its costs. Thus, for each response we chose the value that caused the naïve and the primed population to be of equal size at a specific point in time ${t_\mathrm{ B}}$ and for given costs ${c_{\mathrm{ PB}}}$. We used this baseline scenario as starting point for further analyses on the impacts of stress characteristics and the costs associated with preparation for priming. Since organisms might exhibit enhanced stress responses that are a combination of the earlier, faster or stronger responses, we additionally performed an analysis of stress responses that combine these strategies (see Section 3, Supporting Information).

First, we analytically solved our model to assess the effect of stress duration $\mathrm{ TD}$ and priming costs ${c_\mathrm{ P}}$ on the altered performance of the primed population, given as relative change in population size compared to the naïve population (for calculations, see Section 1, Supporting Information). Based on these results we evaluated whether a naïve response or an earlier, stronger or faster response, respectively, is most beneficial for a population at the end of the applied triggering stress (${t_{\mathrm{ TE}}}$).

To investigate the effect of stress predictability on the benefit of primed response strategies, we analytically assessed different probabilities for a priming cue to correctly predict the occurrence of a triggering stress. Our analysis followed the approach by Mitchell and Pilpel (2011) and is given in Section 4 (Supporting Information).

### Stochasticity in population performance

If the modeled population is of small size, e.g. after encountering a strong stress, additional stochastic fluctuations might drive the population toward extinction. To determine the likelihood of such stochastic extinction events, we formulated the deterministic ODE model as a stochastic model using the Gillespie stochastic simulation algorithm (Pineda-Krch 2010). This requires a numerical solution of the model. For parameterization, we assumed a default growth rate of $g\ = \ 0.0488{t^{ - 1}}$, which corresponds to an approximate growth of 5% per time step. The default mortality is ${m_\mathrm{ I}} = \ 0.0976{t^{ - 1}}$, which is double the growth rate and thus leads to a decrease of 5% per time unit (satisfying ${g_\mathrm{ I}} - \ {m_\mathrm{ I}} = - {g_\mathrm{ I}}\ $). The default response lag $L$ is set to 5$t$, and the time point ${t_\mathrm{ R}}$, at which the final stress response level is reached, is set to 25$t + L$ to allow for stress durations shorter or longer than the buildup of the stress response. The response speed ${s_\mathrm{ R}}$ is set accordingly, i.e. to fulfill ${t_\mathrm{ R}} = \ L + \ ({m_\mathrm{ I}} - {m_\mathrm{ R}})/({m_\mathrm{ I}}{s_\mathrm{ R}}) = \ 30t,$ assuming a final response level of ${m_\mathrm{ R}} = 0.25 \cdot {m_\mathrm{ I}}$. A parameter overview is given in Table 1, and a more detailed investigation of the sensitivity of the ODE model results toward this choice is part of Section 1 (Supporting Information). For the baseline scenario, we applied an intermediate stress duration of $\mathrm{ TD}_\mathrm{ B} = \ 75t$ and priming costs of ${c_{\mathrm{ PB}}} = \ 0.3$. To our knowledge, there are no studies quantifying the costs a priming stimulus exerts on microbes, so we applied moderate costs based on the costs found by Hulten *et al*. (2006), who observed a growth reduction of around 27% in *Arabidopsis* plants primed with β-aminobutyric acid. These costs are of the same magnitude as assumed in a population model of microbes experiencing stress of Mitchell and Pilpel (2011).

**Table 1.** Parameter description and values for a nonprimed population.

Parameter	Description	Default value	Unit
${g_\mathrm{ I}}$	Initial growth rate	0.0488	1/*t*
$K$	Environmental capacity	10 000	^a^
${c_\mathrm{ P}}$	Priming costs	Varied	–
${s_\mathrm{ R}}$	Response speed	0.03	1/*t*
${m_\mathrm{ I}}$	Initial mortality induced by the triggering stress	0.0976	1/*t*
${m_R}$	Minimal mortality reached by the stress response	$0.25 \cdot {m_\mathrm{ I}}$	1/*t*
${t_\mathrm{ R}}$	Time when ${m_\mathrm{ R}}$ is reached	60	*t*
$L$	Lag phase duration	5	*t*
${t_\mathrm{ L}}$	End of lag phase	35	*t*
${t_\mathrm{ P}}$	Beginning of the priming phase	30	*t*
${t_{\mathrm{ TS}}}$	Beginning of the triggering stress	50	*t*
$\mathrm{ TD}$	Stress duration	Varied	*t*
${t_{\mathrm{ TE}}}$	End of the triggering stress and time point of comparison between strategies	Varied	*t*
${c_{\mathrm{ PB}}}$	Priming costs of the baseline scenario, when all three response strategies grant a benefit equal to the naïve response	0.3	–
${t_\mathrm{ B}}$	Time point of the baseline scenario, when all three response strategies grant a benefit equal to the naïve response	75	*t*

^a^ The units are system dependent, e.g. biomass (mg) or colony forming unit.

To implement the stochastic simulation algorithm, we followed the original method (direct method) of Gillespie (1977) and implemented a population of which each unit (e.g. cell) has a certain probability to replicate (${p_1}$) or to die (${p_2}$):

(4) \begin{eqnarray*}
{p_1} = \ g \cdot S,
\end{eqnarray*}

(5) \begin{eqnarray*}
{p_2} = \left\{ {\begin{array}{@{}*{2}{l}@{}} {\left( {\left( { - {s_\mathrm{ R}} \cdot \left( {t - {t_\mathrm{ L}}} \right) + 1} \right) \cdot \ {m_\mathrm{ I}}} \right) \cdot S,}&{t < {t_\mathrm{ R}},}\\ {{m_\mathrm{ R}} \cdot S,}&{t \ge {t_\mathrm{ R}}.} \end{array}} \right.
\end{eqnarray*}

Because we only implemented the phase of stress (${t_{\mathrm{ TS}}} \le t \le {t_{\mathrm{ TE}}}$), priming costs are realized as different initial values of $S$, i.e. ${S_{\mathrm{ np}}}( {t\ = {t_{\mathrm{ TS}}}\ } ) > {S_\mathrm{ p}}( {t\ = {t_{\mathrm{ TS}}}\ } )$, which are parameterized to match the difference after the priming phase in the deterministic model with priming costs of ${c_\mathrm{ P}} = \ 0.3$. The stochastic model was developed and assessed with the R package ‘GillespieSSA’ Version 0.5-4 (Pineda-Krch 2008, 2010).

To assess the effect of stress intensity and stress duration on stress survival, we systematically varied separately the initial mortality ${m_\mathrm{ I}}$ and the stress duration $\mathrm{ TD}$, simulated 10 000 runs of each response strategy and compared which of the different strategies was most beneficial in preventing the population from going extinct. For each response strategy, we recorded the extinction probability as a fraction of runs with population extinction.

### Species and species interactions under resource limitation

For introducing species competition, we extended the original model by resource limitation expressed by the environmental capacity $K$ leading to logistic growth of a population:

(6) \begin{eqnarray*}
\frac{{\mathrm{ d}S}}{{\mathrm{ d}t}} = \ \ g\left( {P,t} \right) \cdot S\left( t \right)\ \cdot \left( {1\ - \ \frac{{S\left( t \right)}}{K}} \right) - m\left( {T,t} \right) \cdot S\left( t \right).
\end{eqnarray*}

First, we numerically investigated the effect of different priming costs and stress durations on the benefits of the three response strategies of a single population. We used the parameter values defined earlier and the assumption of $K\ = \ 10\,000$ and chose the baseline scenario for ${c_\mathrm{ P}} = \ 0.3$ and $\mathrm{ TD} = 75t$. The numerical analysis of all ODEs was performed with the R package ‘deSolve’ Version 1.21 (Soetaert, Petzoldt and Setzer 2010).

Afterward, we investigated whether the optimal stress response shifts under competition. For this, we run simulations of communities containing four microbial populations, each population following one of the four analyzed stress responses: one population was naïve, and the other three populations showed an earlier, a faster or a stronger primed response. Competition between populations was included by a generalized Lotka–Volterra model (Smale 1976). For this, the model of a single population under resource limitation (equation (6)) was expanded by an interaction parameter $\alpha \ \in [ {0,1} ]$, which describes the strength of competition, and by a joint carrying capacity $K$ for all four populations. Each population *S_i_* of the community was then described as

(7) \begin{eqnarray*}
\frac{{\mathrm{ d}{S_i}}}{{\mathrm{ d}t}} = g\left( {P,t} \right) \cdot {S_i}\left( t \right) \cdot \left( {1\ - \ \frac{{{S_i}\left( t \right) + \alpha \cdot \mathop \sum \nolimits_{j \ne i} {S_j}\left( t \right)}}{K}} \right) - m\left( {T,t} \right) \cdot {S_i}\left( t \right).\nonumber\\
\end{eqnarray*}

Similar to the simulations of populations in isolation, we applied a mild priming stimulus and a subsequent strong triggering stress to the community and applied the same set of default parameters. We systematically varied the interaction parameter $\alpha $ between $\alpha \ = \ 0$ (no competition, i.e. same equation as in single-species case) and $\alpha \ = \ 1$ (high competition intensity).

## RESULTS

### Comparison of the three stress responses

**Figure 2.**
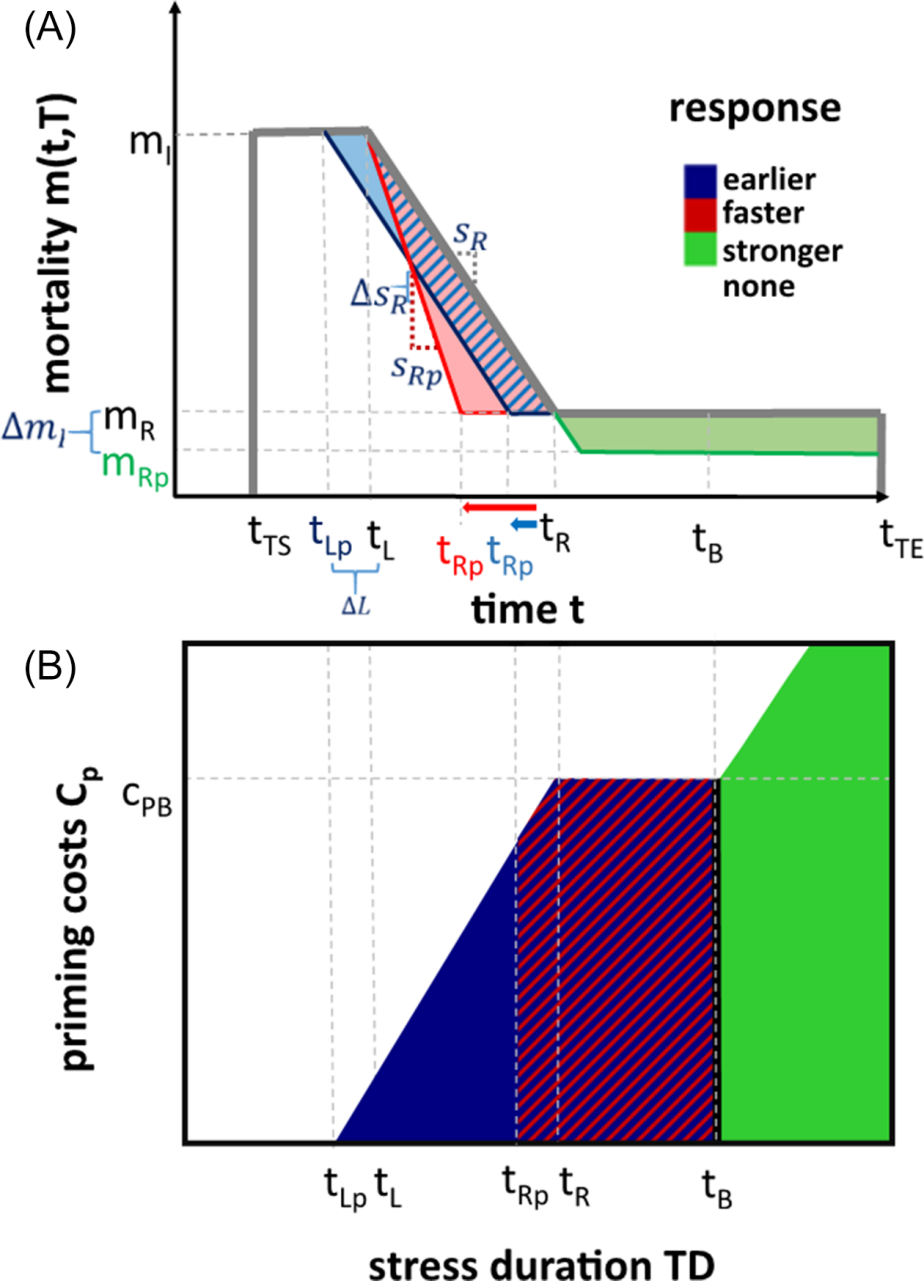
(**A**) Mortality reduction of the three strategies and (**B**) analytically determined parameter space favoring the different primed stress responses under exponential growth depending on stress duration and priming costs. Abbreviations: ${c_{\mathrm{ PB}}}$ = (baseline) priming costs for which the three responses grant a benefit equal to the naïve response; ${m_\mathrm{ I}}$ = initial mortality; ${m_\mathrm{ R}}$ = final mortality level of the stress response; ${m_{\mathrm{ Rp}}}$ = primed (reduced) ${m_\mathrm{ R}}$ (stronger response); ${t_{\mathrm{ TS}}}$ = beginning of the triggering stress; ${t_\mathrm{ L}}$ = end of the response lag phase; ${t_{\mathrm{ Lp}}}$ = end of the primed (shorter) response lag phase (earlier response); ${t_\mathrm{ R}}$ = time point when ${m_\mathrm{ R}}$ has been reached; ${t_{\mathrm{ Rp}}}$ = time point when ${m_\mathrm{ R}}$ has been reached with an earlier or faster stress response; ${t_\mathrm{ B}}$ = (baseline) stress duration when the three responses grant a benefit equal to the naïve response; ${t_{\mathrm{ TE}}}$ = end of triggering stress; ${s_\mathrm{ R}}$ = slope of the mortality reduction; ${s_{\mathrm{ Rp}}}$ = primed (higher) slope of the mortality reduction (faster response)

We analytically assessed which of the three primed response strategies is most beneficial for different stress durations and costs associated with priming (Fig. 2). For short stress durations (${t_{\mathrm{ TE}}} < \ {t_\mathrm{ R}}$), the earlier response is most beneficial, because an early buildup of defense already grants a benefit while other response strategies are still delayed. However, this advantage is compensated for by the faster response for stress durations that are longer than the defense buildup (${t_{\mathrm{ TE}}} \ge \ {t_\mathrm{ R}}$), since both responses reach the same benefit when the final response level ${m_\mathrm{ R}}$ has been reached, i.e. at time point ${t_{\mathrm{ Rp}}}$. For both response strategies, only priming costs of the baseline scenario can be balanced; i.e. for priming costs higher than ${c_{\mathrm{ PB}}}$, priming is not beneficial. Higher costs than those of the baseline scenario will lead to a decrease in performance and higher stress durations cannot compensate that decrease, because after ${t_\mathrm{ R}}$ both responses do not confer increased growth rate compared to the naïve response. The stronger stress response is the most beneficial response for long stress events (stresses that last longer than our defined baseline scenario $\ {t_{\mathrm{ TE}}} \ge \ {t_\mathrm{ B}}$). The longer the stress, the larger the difference in the integral of the stronger stress response compared to the other ones and thus the overall fitness. However, for the stronger stress response, there is no benefit for stress durations shorter than ${t_\mathrm{ R}}$, independent of the costs. This is so because the advantage of the stronger response starts only when the final stress response level ${m_\mathrm{ R}}$ is reached, i.e. at ${t_R}$ (Fig. 1C). For longer stress durations, the benefit increases linearly (green shaded area of Fig. 1C), allowing also for priming costs higher than those of the baseline scenario.

Although the performance of each response type decreases with increasing costs, the fitness rank of the three response types, i.e. which one is most beneficial, is not altered, i.e. priming costs do not affect which response is most beneficial. Moreover, our analytical results show that all response parameters affect population fitness independently of initial mortality ${m_\mathrm{ I}}$ or growth rate $g$; thus, the results are robust to different intensities of stress and different growth conditions. None of the response parameters influences the qualitative pattern of Fig. 2 (see Section 1, Supporting Information), while the shape of the region can vary: a generally faster response (i.e. higher ${s_\mathrm{ R}}$) leads to a reduced value of ${t_\mathrm{ R}}$ and a smaller parameter space favoring only the early response in Fig. 2.

### Stochasticity

**Figure 3.**
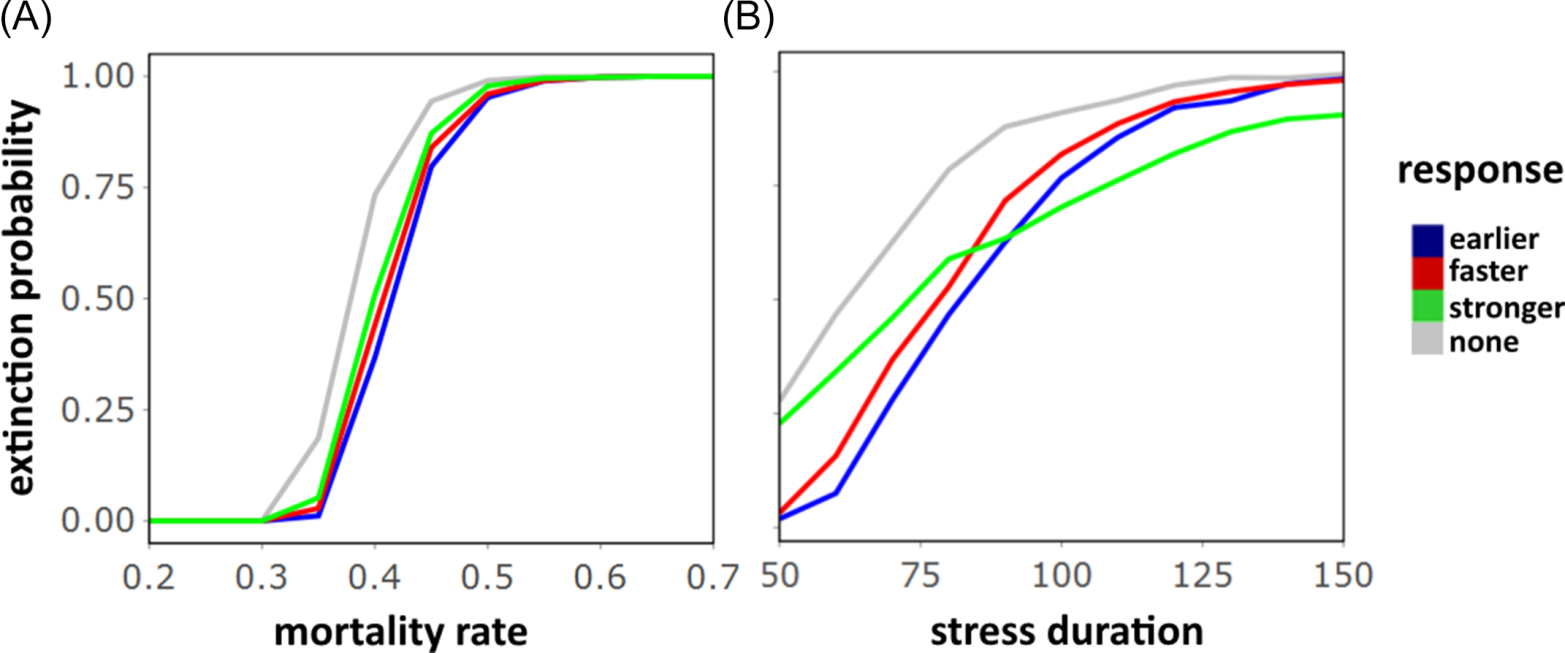
Extinction probability of a population following no or one of the three primed response strategies under (**A**) different stress intensities with stress duration $\mathrm{ TD}\ = \ 75\ $ or (**B**) different stress durations with stress intensity ${m_\mathrm{ I}} = \ 0.4$. The extinction probability is approximated by the fraction of 10 000 populations that did not survive until ${t_{\mathrm{ SE}}}$ (end of stress).

We evaluated the likelihood that a microbial population following none or one of the three different priming strategies becomes extinct under different stress durations and intensities (Fig. 3). The stochastic simulation approach shows that independent of the mortality rate, all primed responses show a decreased extinction probability compared to the naïve stress response. The reduction in extinction risk is of a robust order across all mortality rates (Fig. 3A), with the earlier response providing the lowest risk of extinction, followed by the faster and stronger response. Since the early response decreases the mortality earlier than the other responses, it reduces the risk of driving the population size close to zero, thus reducing extinction probability. The stronger response is less beneficial, as it takes effect later than the other strategies. This pattern changes for longer durations (Fig. 3B): longer exposure to a possibly lethal stress dramatically increases the probability of extinction under a faster and earlier stress response, but does less so under a stronger response strategy. Once the stronger response level ${m_{\mathrm{ Rp}}}$ is reached, this strategy leads to a lower mortality level and a fitness benefit toward the other response strategies.

### Resource limitation and competition

**Figure 4.**
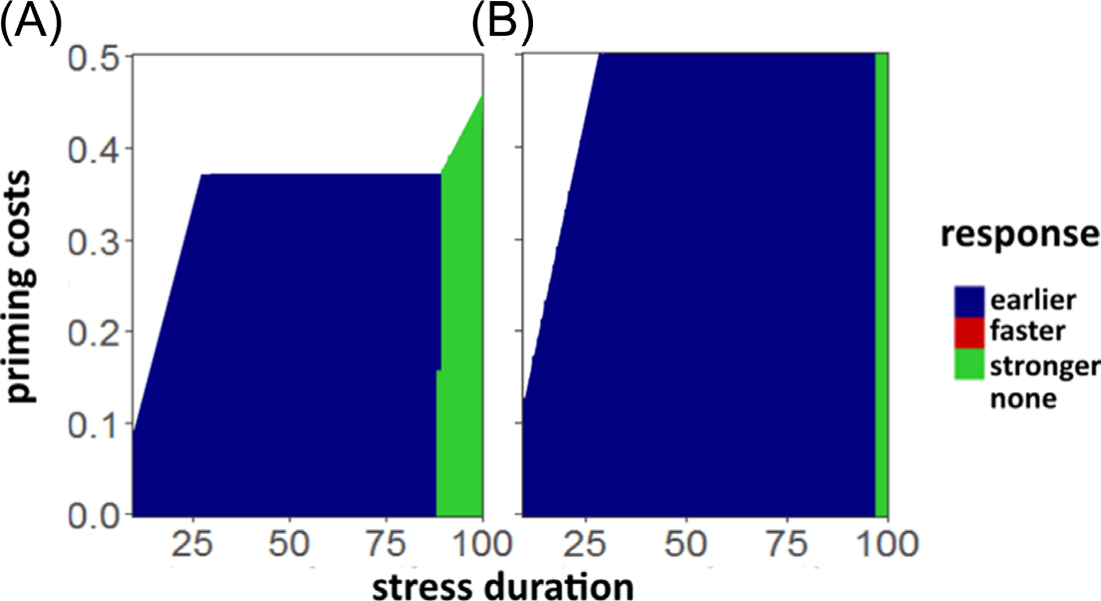
Most beneficial primed response under competition dependent on stress duration and priming costs. Results are given for (**A**) medium competition intensity (${\rm{\alpha \ }} = {\rm{\ }}0.5$) and (**B**) high competition intensity (${\rm{\alpha \ }} = {\rm{\ }}1$).

Finally, we quantified the effect of resource limitation on the benefit of priming for populations in isolation (Fig. S1, Supporting Information) and in the community context (Fig. 4). Under the influence of a limiting carrying capacity $K$, the benefit of the faster response exceeds the earlier response toward the end of the response buildup ${t_\mathrm{ R}}$, as opposed to an equal benefit without $K$ (Fig. S1, Supporting Information). As for the unlimited resources scenario, the stronger response is beneficial for longer durations of stress. The increased benefit of the faster response is caused by the additional density-dependent pressure on the population caused by $K$: The slower (but earlier) buildup of the early response leads to a longer phase where the population following the early response strategy is of increased size, thus subject to increased resource limitation. The faster response, however, exhibits the same reduction of mortality as the earlier response in a shorter amount of time, thus suffering less from resource limitation imposed by the carrying capacity $K$. Competition for resources between response strategies generally leads to higher benefits of priming, as priming is beneficial even for higher priming costs compared to the isolated case (Fig. 4). Moreover, the earlier response outcompetes the faster response; i.e. for the evaluated scenarios, the faster response is never the most beneficial one. For the stronger response to be most beneficial, the stress has to be of longer duration compared to the single-species case, because the benefit of the early response is outperformed later in time. For low competition (Fig. 4A), the overall parameter space of stress duration and priming costs that benefits priming is smaller than that for strong competition, and for strong competition (Fig. 4B), the early primed response grants the highest benefit for most costs/durations, while the stronger response is only more beneficial for a very long stress duration. A visualization of the population dynamics in a community is given in Fig. S2 (Supporting Information).

## DISCUSSION

We used a simple ODE model to assess the benefits of primeability for microbes showing an earlier, faster and stronger stress response than naïve organisms dependent on different scenarios. In the first part of the discussion, we focus on the three primed stress response strategies, and extend the discussion to the effects of stress intensity and growth on our results. In the last part, we discuss the priming response types under resource limitation and competition.

### Benefits of the three primed stress responses

Our analyses show that the duration of stress has a strong impact on which priming strategy might grant the highest benefit, as hypothesized in the introduction. For short and medium duration of stress, the earlier and faster stress responses are most beneficial. This is in accordance with Douma *et al*. (2017), who analyzed primed responses of the plant *Brassica nigra* suffering from herbivory also using a modeling approach. However, their plant model does not account for a lag phase in the response; therefore, we additionally find a benefit of an earlier response for short durations of stress compared to a fast response. In contrast to our assumptions, they associate a stronger stress response with additional defense costs to account for the maintenance during the stress. Implementing additional maintenance costs is reasonable for many forms of defense, but might not apply to all stress defense strategies (e.g. increased production of constitutive defense compounds after wounding in fungi; Spiteller 2008). We thus neglected additional costs of the different responses. However, as long as the maintenance costs are lower than the growth benefit gained by the stronger response, there will always be a net performance gain for longer stress durations, producing results that are qualitatively the same. Another assumption of the model is that the elevated level of the stronger response is maintained as long as the stress lasts, leading to a linearly increasing benefit with stress duration. If we, however, reduced the primed response level back to the naïve response level ${m_\mathrm{ R}}$ at a specific point in time (e.g. because of increased gene expression leveling off or degradation of excess defense molecules), the benefit would not further increase. If this point in time was after until $t > {t_\mathrm{ B}}$, i.e. the time point when the benefit of the faster and earlier responses is compensated for, the stronger response would still be the most beneficial strategy for longer responses, and results would not change qualitatively.

We found that priming costs do not exhibit control over which stress defense type is most beneficial. This is so because the costs affect all response types in the same way, leading to the same decrease in the benefit of priming for all types. For a given stress duration, however, only a certain amount of priming costs can be compensated for, and if costs are too high or the stress duration is too short, it is more profitable not to invest into any type of priming. Here, we implement priming costs as costs that are directly linked to the buildup of the preliminary stress response and do only occur after a priming stimulus. Still, successful priming also requires more general investment in certain mechanisms, for example, the retention of information about a past stress stimulus. Potential memory mechanisms have been discussed in different organisms, such as yeasts (Acar, Becskei and Van Oudenaarden 2005; Zacharioudakis, Gligoris and Tzamarias 2007; Guan *et al*. 2012), prokaryotes (Casadesús and D'Ari 2002; Wolf *et al*. 2008; Lambert *et al*. 2014) and filamentous fungi (Andrade-Linares, Veresoglou and Rillig 2016). This investment constitutes additional costs of priming, which might reduce its overall benefit but affect the three primed responses equally and are thus not expected to change the observed pattern. The effect of memory and a decrease of the primed defense over time could be implemented in our model, e.g. by assuming a linear relationship between the decay or dilution of primed proteins and a reduction of the primed defense level. Assuming that the three response strategies are equally reduced in their efficacy by decreasing memory, the qualitative results of this study, i.e. which response is most beneficial, would still hold. If, however, one of the primed response strategies was associated with a shorter memory of the priming event than the other stress responses, it might lose its benefits. As the underlying physiological processes of response strategies are very diverse, we cannot make a general assumption on whether one primed response strategy exhibits a more sustained memory than another.

### The effect of different stress intensities

In our analyses, we assess the impacts of stress implemented as increased mortality on microbes. Stressors of different intensity can be simulated by our model by assuming different mortality rates ${m_\mathrm{ I}}$: Intense or multifactorial forms of stress, such as fungivory (Döll *et al*. 2013; Ortiz, Trienens and Rohlfs 2013) or low pH (Koutsoumanis and Sofos 2004), lead to the destruction of biomass and can be realized by ${m_\mathrm{ I}} > {g_\mathrm{ I}}$, i.e. an overall decrease of the population size. However, also moderate stress that does not reduce biomass of a population but instead lowers growth can be implemented by values of ${g_\mathrm{ I}} \cdot ( {1 - {c_\mathrm{ p}}} ) < {m_\mathrm{ I}} < {g_\mathrm{ I}}$, representing moderately damaging stresses, while ${m_\mathrm{ I}} = {g_\mathrm{ I}}\ $ would simulate growth halting stress. This particular case has, for example, been shown for hydrogen peroxide concentrations as high as 20 mM, which exhibited a fungistatic and not fungicidal effect on *Metarhizium anisopliae* (Rangel, Anderson and Roberts 2008).

Our model suggests that the stress intensity does not influence the benefit of the priming responses, as the resulting response pattern is the same (see the Supporting Information for an analytical investigation of the effect of stress intensity ${m_\mathrm{ I}}$ on the primed responses). While the intensity of the stress can affect whether priming at all would pay off, which of the three responses is most beneficial depends on how much time the organisms has to build up a stress response. However, the observed pattern changes for stress intensities that are high enough to drive a population to extinction. Under the pressure of a possibly lethal stress, the relative benefit of the early stress response increases, because it is the first response to take effect and thus most likely to prevent the population from dying out. If a severe stress is of longer duration and a population survives the initial one, the stronger response pays off, as it reduces the stress impact further than the other responses and increases chances for survival.

### Priming under resource limitation and competition

If we introduce a carrying capacity $K$ into the model, the benefit of the faster response increases and surpasses the earlier response for intermediate stress durations. Here, the advantage of the faster response is an increase in defense in a relatively shorter amount of time compared to the earlier response, which leads to a more efficient exploitation of the environmental limitations. Because the final response level and the resulting mortality are the same for both responses, the benefit of both strategies converges for longer stress durations. However, this benefit shifts under competition between species: We found that the community context can alter the costs and benefits of induced defenses, as the benefit of priming is increasing and even high costs can be compensated for. This is in line with the results of Rillig *et al*. (2015), who found that competition enhances the payoff arising from priming. Under competition, the early response leads to a priority effect (Kennedy, Peay and Bruns 2009) and thus provides a larger benefit than in the single-species context: A population reacting earlier to a stress can acquire nutrients and space before the population following a different response type has started building up its defense. Therefore, even for low competition (expressed by a low value of *α*; see equation (6)), the early response outperforms the faster response in all cases. For a better understanding of the underlying community dynamics, we added two timelines of community development to Fig. S2 (Supporting Information). The stronger the competition between species (high value of *α*), the higher the benefits of priming. Therefore, in the community context priming is beneficial under higher costs than for isolated species. As shifts in the composition of microbial communities after disturbance are common (Schimel, Balser and Wallenstein 2007), priming might not only influence the short-term physiological responses of the community members, but also the overall composition of a community. Favoring primeable species following a certain strategy more than others, priming could thus change the effect of disturbance legacy (Jurburg *et al*. 2017) and have a long-lasting effect on ecosystem process rates and community function (Allison and Martiny 2008).

### Priming uncertainty and combination of different priming responses

Our results are based on the assumption that the priming cue predicts the upcoming stress without error. In reality, however, the benefit of priming will be greatly reduced by the degree of the predictability of the disturbance (Mitchell and Pilpel 2011; Katz and Springer 2016; Douma *et al*. 2017). Therefore, we analytically investigated the effect of predictability on the benefit of primed response strategies. While predictability influences whether priming at all is beneficial compared to the naïve response, it does not impact which of the three priming response types is the most beneficial for a given stress duration; i.e. the observed pattern is robust even under unpredictable environments (see the Supporting Information).

So far, we have only discussed the primed response types under the assumption that they are mutually exclusive. It is to be expected, however, that organisms exhibit mixed responses to increase their defense. Soil fungi that were temperature primed and exposed to severe heat, for example, showed an earlier regrowth and higher overall growth than naïve fungi (Andrade-Linares, Veresoglou and Rillig 2016). We analytically investigated the benefit of primed responses incorporating two strategies and showed that for shorter durations, the combination of fast and early, while for longer stress durations the fast and strong primed response is most beneficial (see Section 3 and Fig. S1b, Supporting Information), because with a faster buildup, the final response level ${m_\mathrm{ R}}$ is reached quickly and the stronger benefit takes effect earlier.

Our theoretical approach has provided novel insights into the benefits of different priming responses dependent on species traits, such as specific priming costs, and stress characteristics, i.e. the stress intensity and duration. Although the level of abstraction in our model approach is high, we could relate the findings to empirical studies and propose which priming responses are most beneficial and thus most likely to find under a given set of conditions. We could show that the stronger primed response is most beneficial for longer stress phases, while the faster and earlier responses increase performance under short durations of stress. More fatal levels of stress that might drive populations to the edge of extinction are best met with early defense strategies. Thus, at the ecological level, the dynamics of priming can be highly variable and the benefits of different priming responses depend strongly on abiotic and biotic environmental factors. We therefore expect to find different priming responses to co-occur under varying stress conditions, while a more homogeneous stressor (e.g. in terms of stress duration) might favor similar priming strategies across populations. That is, we hypothesize primed stress responses to be more diverse under diverse stressors.

Priming in the community context has a higher significance than in isolation, and disturbance (i.e. a trigger stress) will benefit certain primed response strategies stronger than other strategies, thus shifting community composition. In systems prone to frequent disturbances and a high degree of competition, timing of colonization is vital and the early primed response is most beneficial. For systems experiencing longer stress durations, the stronger response gains in significance.

With our study, we would like to stimulate a discussion of priming effects that goes beyond the molecular basis of priming, but that considers priming in the ecological context. Our work shows that priming effects vary between the community context and between stress characteristics, but that some patterns are robust across environmental settings. These theoretical findings now need to be complemented with empirical studies and should find their way into stress ecology in general.

## Supplementary Material

fiz114_Supplemental_Files
