# Supplementary material for: Primed to be strong, primed to be fast: modeling benefits of microbial stress responses
Source: FEMS Microbiol Ecol. 2019 Jul 11;95(8):fiz114. doi: 10.1093/femsec/fiz114 (PMC6657816; doi:10.1093/femsec/fiz114)

**Supporting Information**

Article Title: Primed to be strong, primed to be fast: modelling benefits of microbial stress responses

Authors: Felix Wesener, Britta Tietjen

1. **Parameter analysis**

To determine the effect of the parameter choice for our baseline scenario, we analytically investigated the benefit of priming under different parameter sets. To do so, we solved the differential equation described by Equation 1 and assessed the effect of parameter choice on the three different response parameters. Because the mortality $m(t)$ is defined by a step function depending on the stress phase that the organism is currently experiencing, we obtained three different solutions:

$S\left( t \right)=\left\{ \begin{matrix} S_{0}\cdot e^{(g-m_{I})\cdot t}, & t<t_{L} \\ S_{0}\cdot e^{\left( g-m_{I} \right)\cdot t+\frac{{(L}^{2}+t^{2})(m_{I}s_{R})}{2}-tLm_{I}s_{R}}, & t_{L}\leq t<t_{R} \\ S_{0}\cdot e^{\frac{m_{I}s_{R}t_{R}^{2}}{2}-Lm_{I}s_{R}t_{R}+m_{R}t_{R}-m_{I}t_{R}+\frac{L^{2}m_{I}s_{R}}{2}-tm_{R}+gt}, & t\geq t_{R} \end{matrix} \right.$ (S1)

with $t_{R}=L+\frac{m_{I}-m_{R}}{m_{I}s_{R}}$: (S2)

$S\left( t \right)=\left\{ \begin{matrix} S_{0}\cdot e^{(g-m_{I})\cdot t}, & t<t_{L} \\ S_{0}\cdot e^{\left( g-m_{I} \right)\cdot t+\frac{{(L}^{2}+t^{2})(m_{I}s_{R})}{2}-tLm_{I}s_{R}}, & t_{L}\leq t<t_{R} \\ S_{0}\cdot e^{\left( g-m_{R} \right)\cdot t+\left( m_{R}-m_{I} \right)\cdot L+\frac{2m_{I}m_{R}-m_{I}^{2}-m_{R}^{2}}{2m_{I}s_{R}}}, & t\geq t_{R} \end{matrix} \right.$ (S3)

For the baseline scenario, we define the primed and nonprimed population as being of equal size at time point $t_{B}$, i.e. $S_{p}\left( t=t_{B} \right)=S_{np}\left( t=t_{B} \right)$. The parameters determining the improved primed response thus compensate the priming costs that initially lead to a higher population size of the nonprimed population at the beginning of the stress $S_{p}(t=t_{TS})<S_{np}(t=t_{TS})$. These improved response parameters are either a shorter length of the primed lag phase $L_{p}$ (earlier response), the higher speed of the primed response $s_{Rp}$ (faster response), or the improved final primed response level $m_{Rp}$ (stronger response).

In case of $t_{L}\leq t<t_{R},$ the stress response is building up, and the parameters affect the system dynamics differently than after the response buildup. For this case, only the primed response speed $s_{Rp}$ and the primed lag phase $L_{p}$ are investigated, because the final response level $m_{R}$ has not yet been reached and the primed stronger response cannot show any positive effect. We analytically investigate, which response brings a higher benefit during response buildup and whether the parameter choice influences our results. To do so, we can assess the population size during the response buildup ($t_{L}\leq t<t_{R}$) for the earlier and faster results with:

$S_{p\_early}=S_{p}\cdot e^{\left( g-m_{I} \right)\cdot t+\frac{{((L-\Delta L)}^{2}+t^{2})(m_{I}s_{R})}{2}-t(L-\Delta L)m_{I}s_{R}}$ (S4)

${S_{p\_fast}=S}_{p}\cdot e^{\left( g-m_{I} \right)\cdot t+\frac{{(L}^{2}+t^{2})(m_{I}s_{R\cdot\Delta s_{R}})}{2}-tLm_{I}s_{R}\cdot\Delta s_{R}}$ (S5)

In the following, we will refer to the initial vale of a primed or naïve population as $S_{p}$ or $S_{np}$, respectively. Figure 2 shows that if we define our baseline scenario (i.e. that time point when all three responses confer a benefit as high as the nonprimed response) at any point in time $t>t_{R}$, the earlier and faster response will be of equal benefit at $t\geq t_{R}$, because these responses do not accumulate any additional benefit after the response buildup, unlike the stronger response. We can thus assume $S_{p\_early}\left( t=t_{R} \right)={S_{p\_fast}\left( t=t_{R} \right)=S}_{np}\left( t=t_{R} \right)$ and use our second solution (Eq. S3) to solve for the necessary reduction in the lag phase$\Delta L$ and the increase in speed $\Delta s_{R}$ to compensate for priming costs:

${\Delta L=L-L}_{p}=L-t_{R}+\sqrt{\frac{2ln(\frac{S_{np}}{S_{p}})}{m_{I}s_{R}}+(L-t_{R})^{2}}$ (S6)

$\Delta s_{R}=\frac{s_{Rp}}{s_{R}}=1+\frac{ln(\frac{S_{np}}{S_{p}})}{\left( L-t_{R} \right)^{2}m_{I}s_{R}}$ (S7)

By inserting Equations S6 and S7 into S4 and S5, respectively, we can assess the difference between the relative population sizes $\frac{S_{p\_early}-S_{p\_fast}}{S_{np}}$:

$$S_{p\_early}-S_{p\_fast}=S_{p}\cdot e^{\left( g-m_{I} \right)\cdot t+\frac{{((L-\Delta L)}^{2}+t^{2})(m_{I}s_{R})}{2}-t\left( L-\Delta L \right)m_{I}s_{R}}-S_{p}\cdot e^{\left( g-m_{I} \right)\cdot t+\frac{{(L}^{2}+t^{2})(m_{I}s_{R\cdot\Delta s_{R}})}{2}-tLm_{I}s_{R}\cdot\Delta s_{R}}$$

To find out whether this equation is negative or positive, it is sufficient to investigate only the exponential terms that are different between $S_{p\_early}$ and $S_{p\_fast}$:

$$\frac{{(L_{p}}^{2}+t^{2})(m_{I}s_{R})}{2}-tL_{p}m_{I}s_{R}-(\frac{{(L}^{2}+t^{2})(m_{I}s_{Rp})}{2}-tLm_{I}s_{Rp})=\frac{m_{I}}{2}(\left( \left( t^{2}-2L_{p}t+L_{p}^{2} \right)s_{R}-\left( L^{2}-2tL+t^{2} \right)s_{Rp} \right)=\frac{m_{I}}{2}(\left( t-L_{p} \right)^{2}s_{R}-\left( t-L \right)^{2}s_{Rp})$$

Here, we can focus on two terms. If the left term is larger than the right, then the early population will be larger than the faster.

$$\left( t-L_{p} \right)^{2}s_{R}>\left( t-L \right)^{2}s_{Rp}$$

We can now substitute $L_{p}$ and $s_{Rp}$ from Equations S6 and S7, respectively:

$$\left( t-t_{R}+\sqrt{\frac{2\ln\left( \frac{S_{np}}{S_{p}} \right)}{m_{I}s_{R}}+\left( L-t_{R} \right)^{2}} \right)^{2}s_{R}>\left( t-L \right)^{2}s_{R}+\frac{\ln\left( \frac{S_{np}}{S_{p}} \right)\left( t-L \right)^{2}}{\left( L-t_{R} \right)^{2}m_{I}}$$

We can show that for no priming costs (i.e. $S_{np}=S_{p}$) , both terms are equal:

$$\left( t-L \right)^{2}s_{R}=\left( t-L \right)^{2}s_{R}$$

And, ignoring the additive terms, we can show that costs higher than zero lead to a benefit of the early response:

$$\left( \sqrt{\frac{2\ln\left( \frac{S_{np}}{S_{p}} \right)}{m_{I}s_{R}}} \right)^{2}s_{R}>\frac{\ln\left( \frac{S_{np}}{S_{p}} \right)\left( t-L \right)^{2}}{\left( L-t_{R} \right)^{2}m_{I}}$$

$$2\ln\left( \frac{S_{np}}{S_{p}} \right)>\frac{\left( t-L \right)^{2}}{\left( t_{R}-L \right)^{2}}\ln\left( \frac{S_{np}}{S_{p}} \right)$$

$$2>\frac{\left( t-L \right)^{2}}{\left( t_{R}-L \right)^{2}}$$

$S_{p\_early}$ > $S_{p\_fast}$ for all $t<t_{R}$ and any value of $m_{I},s_{R},L$, $m_{R}$, or $g$. Thus, at any point during the response buildup, the earlier response is more beneficial than the faster response due to the earlier setoff. This benefit is compensated for by the faster response only later in time when the buildup has been finished. We can now assess the impacts parameter choice on our results: A faster response buildup (higher $s_{R}),$ a shorter lag phase $L$ or a higher final response level $m_{R}$ will decrease the value of $t_{R}$ (Eq. S2 and Fig. 2) and the response buildup is finished earlier. An increase in stress intensity $m_{I}$ reduces the strength of the primed responses $\Delta L$ and $\Delta s_{R}$ (Eq. S6 and S7, respectively), because under more intense stress the same priming costs can be compensated for with less investments. The maximum difference between the benefit of early and fast response during the response buildup is higher for higher $m_{I}$ due to the higher benefit gain of the early response during the response lag $t<t_{L}$.

In case of $t \geq t_{R}$, the final response level has been reached, and the third solution of Equation S3 describes the population dynamics. Again, we use the time point $t_{B}$ as reference point at which nonprimed and primed population are of equal size $S_{p}\left( t=t_{B} \right)=S_{np}\left( t=t_{B} \right)$, with $t_{R}<t_{B}$ (Fig. 2).

$S_{p\_early}=S_{p}\cdot e^{\left( g-m_{R} \right)\cdot t+\left( m_{R}-m_{I} \right)\cdot(L-\Delta L)+\frac{2m_{I}m_{R}-m_{I}^{2}-m_{R}^{2}}{2m_{I}s_{R}}}$ (S8)

${S_{p\_fast}=S}_{p}\cdot e^{\left( g-m_{R} \right)\cdot t+\left( m_{R}-m_{I} \right)\cdot L+\frac{2m_{I}m_{R}-m_{I}^{2}-m_{R}^{2}}{2m_{I}s_{R}\Delta s_{R}}}$ (S9)

$S_{p\_strong}=S_{p}\cdot e^{\left( g-m_{R}\Delta m_{R} \right)\cdot t+\left( m_{R}\Delta m_{R}-m_{I} \right)\cdot L+\frac{2m_{I}m_{R}\Delta m_{R}-m_{I}^{2}-(m_{R}\Delta m_{R})^{2}}{2m_{I}s_{R}}}$ (S10)

In this phase, the three response parameters are defined as:

${\Delta L=L-L}_{p}=\frac{ln(\frac{S_{np}}{S_{p}})}{m_{I}-m_{R}}$ (S11)

$\Delta s_{R}=s_{Rp}/s_{R}= \frac{(m_{R}-m_{I})^{2}}{-2\ln\left( \frac{S_{np}}{S_{p}} \right)m_{I}s_{R}+(m_{R}-m_{I})^{2}}$ (S12)

$\Delta m_{R}={m_{R}-m}_{Rp}=-\frac{ln\left( \frac{S_{np}}{S_{p}} \right)}{t_{R}-t_{B}}$ (S13)

We can now compare the population size for any $t\geq t_{R}$ with equations S8-S10, and the response parameter values derived in Equations S10-S12. We can show that $S_{p\_early}=S_{p\_fast}$ for all $t>t_{R}$, independently of any other parameters:

$$S_{p_{early}}=S_{p_{fast}}$$

$$S_{p}\cdot e^{\left( g-m_{R} \right)\cdot t+\left( m_{R}-m_{I} \right)\cdot L_{p}+\frac{2m_{I}m_{R}-m_{I}^{2}-m_{R}^{2}}{2m_{I}s_{R}}}{=S}_{p}\cdot e^{\left( g-m_{R} \right)\cdot t+\left( m_{R}-m_{I} \right)\cdot L+\frac{2m_{I}m_{R}-m_{I}^{2}-m_{R}^{2}}{2m_{I}s_{Rp}}}$$

$$S_{p}\cdot e^{\left( g-m_{R} \right)\cdot t+\left( m_{R}-m_{I} \right)\cdot L+\ln\left( \frac{S_{np}}{S_{p}} \right)-\frac{\left( m_{R}-m_{I} \right)^{2}}{2m_{I}s_{R}}}=S_{p}\cdot e^{\left( g-m_{R} \right)\cdot t+\left( m_{R}-m_{I} \right)\cdot L+\ln\left( \frac{S_{np}}{S_{p}} \right)-\frac{\left( m_{R}-m_{I} \right)^{2}}{2m_{I}s_{R}}}=S_{np}\cdot e^{\left( g-m_{R} \right)\cdot t+\left( m_{R}-m_{I} \right)\cdot L-\frac{\left( m_{R}-m_{I} \right)^{2}}{2m_{I}s_{R}}}$$

$S_{p\_strong}$ is lower than the populations following the other strategies up to $t_{B}$, when all responses are of equal benefit. Afterwards, the stronger strategy further accumulates benefit and is therefore superior to the other strategies. This can be shown by looking at the third solution of Equation S1:

$$S_{p\_strong}=S_{p}\cdot e^{\frac{m_{I}s_{R}t_{R}^{2}}{2}-Lm_{I}s_{R}t_{R}+m_{R}\Delta m_{R}t_{R}-m_{I}t_{R}+\frac{L^{2}m_{I}s_{R}}{2}-tm_{R}\Delta m_{R}+gt}$$

And inserting $\Delta m_{R}$ as defined in Equation S13.

$$S_{p}\cdot e^{\frac{m_{I}s_{R}t_{R}^{2}}{2}-Lm_{I}s_{R}t_{R}+m_{R}t_{R}+\frac{ln\left( \frac{S_{np}}{S_{p}} \right)t_{R}}{t_{R}-t_{B}}-m_{I}t_{R}+\frac{L^{2}m_{I}s_{R}}{2}-tm_{R}-\frac{ln\left( \frac{S_{np}}{S_{p}} \right)t}{t_{R}-t_{B}}+gt}$$

$${=S}_{p}\cdot e^{\frac{m_{I}s_{R}t_{R}^{2}}{2}-Lm_{I}s_{R}t_{R}+m_{R}t_{R}-m_{I}t_{R}+\frac{L^{2}m_{I}s_{R}}{2}-tm_{R}+\frac{ln\left( \frac{S_{np}}{S_{p}} \right)(t_{R}-t)}{t_{R}-t_{B}}+gt}=S_{p}\cdot e^{\frac{m_{I}s_{R}t_{R}^{2}}{2}-Lm_{I}s_{R}t_{R}+m_{R}t_{R}-m_{I}t_{R}+\frac{L^{2}m_{I}s_{R}}{2}-tm_{R}+gt}\cdot e^{\frac{ln\left( \frac{S_{np}}{S_{p}} \right)(t_{R}-t)}{t_{R}-t_{B}}}$$

For $t=t_{B}$, we obtain

$$S_{p\_strong}=S_{p}\cdot e^{\frac{m_{I}s_{R}t_{R}^{2}}{2}-Lm_{I}s_{R}t_{R}+m_{R}t_{R}-m_{I}t_{R}+\frac{L^{2}m_{I}s_{R}}{2}-tm_{R}+gt}\cdot e^{ln\left( \frac{S_{np}}{S_{p}} \right)}=S_{np}\cdot e^{\frac{m_{I}s_{R}t_{R}^{2}}{2}-Lm_{I}s_{R}t_{R}+m_{R}t_{R}-m_{I}t_{R}+\frac{L^{2}m_{I}s_{R}}{2}-tm_{R}+gt}=S_{nonprimed}$$

For all values $t>t_{B}$, the value of $S_{p\_strong}$ will be larger than $S_{nonprimed}$, because $e^{\frac{ln\left( \frac{S_{np}}{S_{p}} \right)(t_{R}-t)}{t_{R}-t_{B}}}$ is growing with $t>t_{R}$.

1. **Effect of resource limitation**

We numerically analyze the differential equation model described by Equation 1 under the assumption of resource limitation, i.e. carrying capacity $K=10,000$. The system is parametrized as described in the Methods section of the main manuscript. We systematically assess the effect of different priming costs and stress durations on the benefit of the three primed stress responses and can show an increased benefit of the faster response for intermediate durations of stress compared to the solution without $K$ (Fig. S1a).

1. **Combined Responses**

Just as the analysis shown above, we can analyze the benefit of two response types combined into one defense strategy, namely a response that starts earlier and builds up faster than a nonprimed response, or a response with a higher defense level that either starts earlier or is realized faster. To realize a combined response and facilitate comparison, we directly transfer the change of response parameters $\Delta L,\Delta s_{R},\Delta m_{R}$ that provide the same benefit at $t=t_{B}$ in the single response case to the combined response case. That is, we do not seek for parameters to make the combined response equal to the nonprimed case or to the single response at $t=t_{B}$, since this would add an additional degree of freedom to the parameter choice. Instead, we compare the three combined responses among each other, focusing on the stress defense phase after the response buildup $t>t_{R}$:

$S_{p\_early\_fast}=S_{p}\cdot e^{\left( g-m_{R} \right)\cdot t+\left( m_{R}-m_{I} \right)\cdot(L-\Delta L)+\frac{2m_{I}m_{R}-m_{I}^{2}-m_{R}^{2}}{2m_{I}s_{R}\Delta s_{R}}}$ (S13)

${S_{p\_fast\_strong}=S}_{p}\cdot e^{\left( g-m_{R}\Delta m_{R} \right)\cdot t+\left( m_{R}\Delta m_{R}-m_{I} \right)\cdot L+\frac{2m_{I}m_{R}\Delta m_{R}-m_{I}^{2}-\left( m_{R}\Delta m_{R} \right)^{2}}{2m_{I}s_{R}\Delta s_{R}}}$ (S14)

$S_{p\_early\_strong}=S_{p}\cdot e^{\left( g-m_{R}\Delta m_{R} \right)\cdot t+\left( m_{R}\Delta m_{R}-m_{I} \right)\cdot(L-\Delta L)+\frac{2m_{I}m_{R}\Delta m_{R}-m_{I}^{2}-(m_{R}\Delta m_{R})^{2}}{2m_{I}s_{R}}}$ (S15)

Here, $S_{p\_early\_fast}>S_{p\_fast\_strong}>$ $S_{p\_early\_strong}$ holds during shorter stress durations after $t_{R}$, while for longer stress durations the combination of a faster buildup and a stronger response is most beneficial ($S_{p\_fast\_strong}>$ $S_{p\_early\_strong}{>S}_{p\_early\_fast})$.

**Figure S1** Parameter space favoring the different primed stress responses under resource limitation depending on stress duration and priming costs; a) single response strategies and b) combined response strategies.


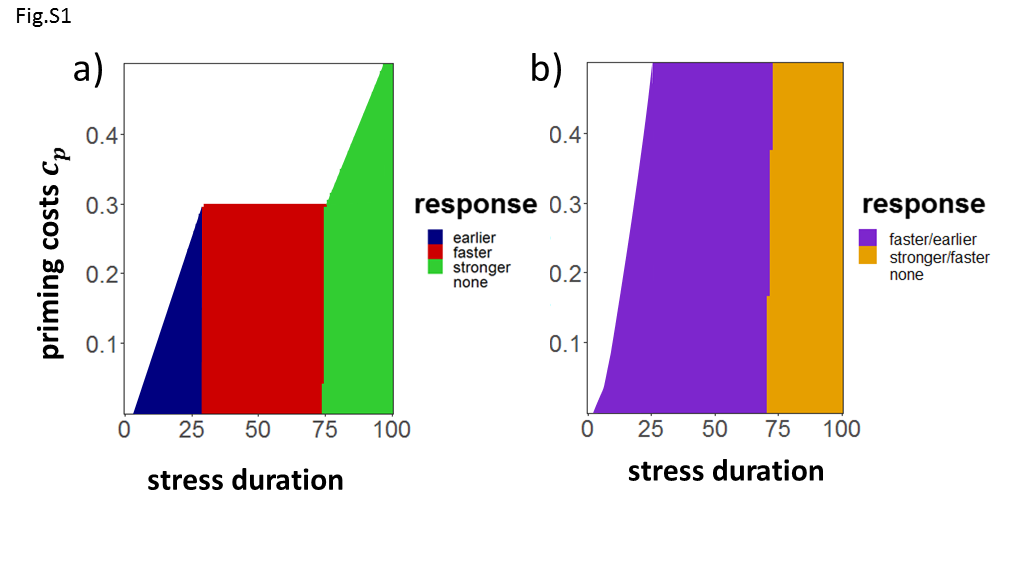


We numerically implemented the combined responses with the same parameter set as the isolated response scenario and a carrying capacity $K=10,000$ and could reproduce the analytical results (see Fig. S1b). The parameter space favoring priming is much larger, because the combined responses yield a higher benefit, compensating the priming costs. However, these more complex responses will most likely come with higher costs.

1. **Predictability of stress**

To approximate the costs of poor predictability of stress, we differentiate two alternative options: If a priming cue is followed by a triggering stress (denoted as $T=True$), it can lead to a fitness gain $\Delta F_{PT}$ that we define as difference between primeable $S_{p}(t)$ and nonprimeable population $S_{np}(t)$.

$\Delta F_{PT}=S_{p}\left( T=True,t=t_{TE} \right)- S_{np}\left( T=True,t=t_{TE} \right)$ (S13)

Note that in our model, $\Delta F_{PT}$ can be negative if the priming costs are high or the stress duration is short. $t_{TE}$ is the time point of measurement just after the triggering stress has ended.

If no triggering stress occurs after an organism has been primed (denoted as $T=False$), the costs of establishing a preliminary priming response have still been paid, but the possible benefit of reacting earlier/faster/stronger to a stress will not be gained and the difference $\Delta F_{P}$ will be calculated as

$\Delta F_{P}=S_{p}\left( T=False,t=t_{TE} \right)- S_{np}\left( T=False,t=t_{TE} \right)$ (S14)

$\Delta F_{P}$ will always be negative (or zero), because without triggering stress, no benefit is gained by committing to a priming strategy and the nonprimed population $S_{np}$ will perform better.

We can now use these values for a given stress duration and given costs of priming to assess the effect of predictability, i.e. the probability $p(T=True)$ that a priming cue is followed by a triggering stress. We calculate the overall fitness by

$\Delta F=p(T=True)\cdot\Delta F_{PT}+(1-p(T=True))\cdot\Delta F_{P}$ (S16)

For a poorly predictable stress, $(1-p(T=True))$ will be higher and the overall Fitness gain $\Delta F$ will be lower, as more priming events are followed by no stress and thus the overall costs invested per successfully predicted triggering stress increase. $\Delta F_{P}$ is independent of the priming responses, because in the case of no triggering stress, no primed stress response occurs. The benefits of the responses occurring in the case of $\Delta F_{PT}$ will thus all be equally reduced by $(1-p(T=True))\cdot\Delta F_{P}$.

1. **Community dynamics**

To better understand the dynamics in the community model and the time point of changes in dominance, a visualization of the population dynamics in a community is given in Figure S2. The early population is dominant because of the earlier reduction of mortality that takes place while the other responses are still delayed in their response. The faster population cannot compensate for this delay, because it shares the same capacity and is limited by the larger population size of the early population. Only for longer stress duration, the stronger population dominates the community because of its smaller mortality, leading to higher overall growth.

**Figure S2** Population sizes of the three primed response strategies under a) short stress ($TD=75$) and b) long stress ($TD=150$) and intermediate competition ($\alpha=0.5$). Population size is relative to the nonprimed strategy (shown as black line), i.e. a value of 1 signifies a population size equal to the naïve population. The Priming stimulus takes place at $t=30$, and the triggering stress begins at $t=50$.


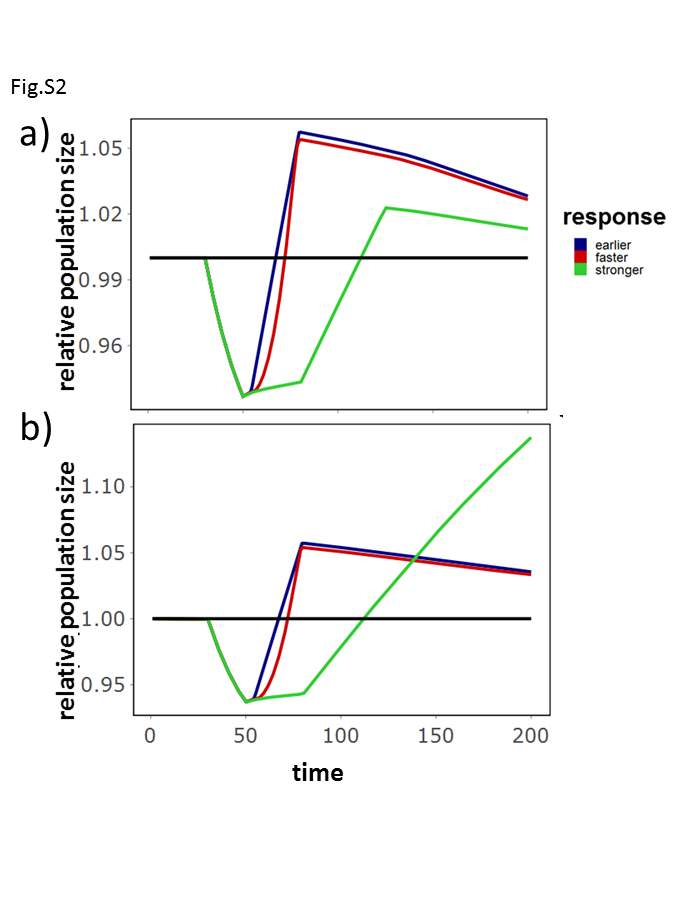

Supplement: fiz114_Supplemental_Files [file fiz114_supplemental_files.zip › Wesener_Tietjen_SI_revised.docx]
