# Supplementary figures and images for: Primed to be strong, primed to be fast: modeling benefits of microbial stress responses
Source: FEMS Microbiol Ecol. 2019 Jul 11;95(8):fiz114. doi: 10.1093/femsec/fiz114 (PMC6657816; doi:10.1093/femsec/fiz114)

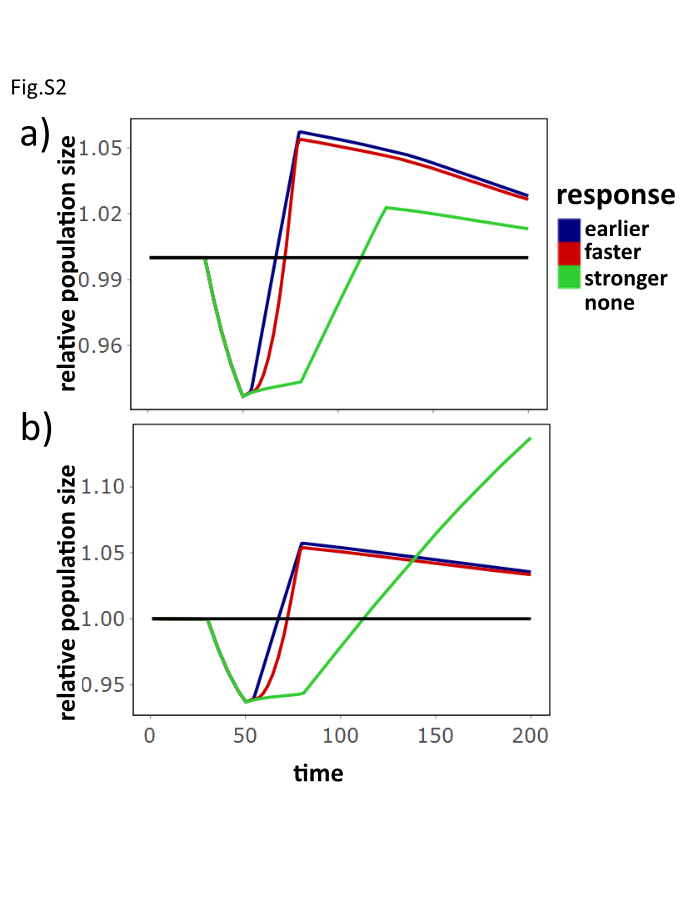

Supplement: fiz114_Supplemental_Files [file fiz114_supplemental_files.zip › figureS2.tiff]

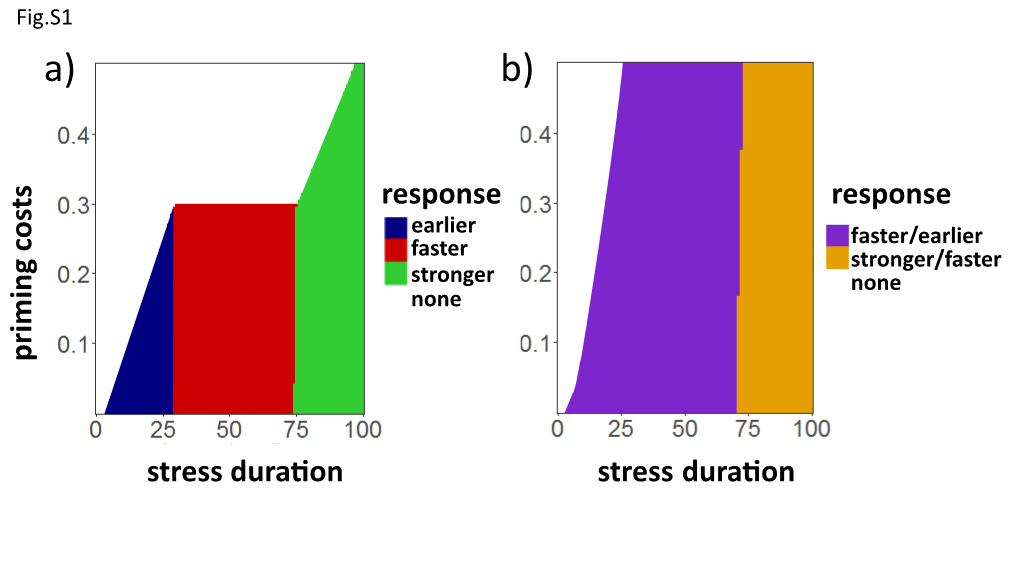

Supplement: fiz114_Supplemental_Files [file fiz114_supplemental_files.zip › figure_S1.tiff]
